# Supplementary material for: Improving community health worker treatment for malaria, diarrhoea, and pneumonia in Uganda through inSCALE community and mHealth innovations: A cluster randomised controlled trial
Source: PLOS Digit Health. 2023 Jun 12;2(6):e0000217. doi: 10.1371/journal.pdig.0000217 (PMC10260253; doi:10.1371/journal.pdig.0000217)
Supplement: S1 File — (DOCX) [file pdig.0000217.s001.docx]

# Scoring instrument for inSCALE CHW Performance

The CHW Performance assessment tool was based on the WHO Health Worker Performance Assessment Instrument (non-observed). The performance of CHWs in treating malaria, diarrhoea and pneumonia both in mild and severe forms were tested by an inSCALE interviewer using theoretical scenarios with sick children. For every unprompted correct action mentioned in response, the interviewer marked the corresponding item on the checklist (see data collection forms).

**S1 File. Item weighting – Case study: Francis/Alberte (pneumonia and diarrhoea)**

| **Item** | **Score weight** | **Item** | **Score weight** | **Item** | **Score weight** |
| --- | --- | --- | --- | --- | --- |
| Asks about the duration of child’s cough | 1 | Prescribes antibiotics; amoxicillin | 1 | Arranges a follow-up visit | 0.5 |
| Asks if child had fever | 0.5 | Prescribes zinc tablets | 1 | Records visit in CHW registration book | 0.5 |
| Asks if any blood in child’s stool | 0.5 | Advises dose of AL/Coartem – 3 days | 0 | Advises continued feeding and fluids for child | 0.5 |
| Asks if child has been vomiting | 0.5 | Advises dose of ORS – as often as needed | 1 | Advises to return or take child to facility if worsens | 0.5 |
| Asks if child had convulsions | 0.5 | Gives first dose of amoxicillin | 1 | Checks child health or vaccination record | 0.5 |
| Ask about the duration of child’s diarrhoea | 1 | Gives first dose of ORS | 1 |  |  |
| Asks about the duration of child’s fever | 0 | Gives first dose of zinc | 1 |  |  |
| Asks/checks whether child can drink/breastfeed | 0.5 | Prescribes anti-malarial; Coartem | 0 |  |  |
| Asks/checks if child is very sleepy/unconsciousness | 0.5 | Prescribes ORS | 1 |  |  |
| Asks if child had any other problem | 0.5 | Advises dose of amoxicillin – 5 days | 1 |  |  |
| Counts breaths of child | 1 | Advises dose of zinc – 10 days | 1 |  |  |
| Performs an RDT (blood test for malaria) on child | 0 | Gives first dose of AL/Coartem | 0 |  |  |
| Checks for chest indrawing | 1 | Gives dose of rectal artesunate/plasmotrim | 0 |  |  |
| Identifies the child as having fast breathing/pneumonia | 1 | Child does not need any treatment from CHW | 0 |  |  |
| Identifies the child as having danger sign/referral symptom or severe illness (any) | 0 | Refers to health facility | 0 |  |  |
| Identifies the child as having malaria | 0 | Writes referral note | 0 |  |  |
| Identifies the child as having diarrhoea | 1 | Advises use of ITN for child | 0.5 |  |  |

**Supplement Table 2. Item weighting – Case study: Hope/Tina (malaria)**

| **Item** | **Score weight** | **Item** | **Score weight** | **Item** | **Score weight** |
| --- | --- | --- | --- | --- | --- |
| Asks about the duration of child’s fever | 1 | Advises dose of AL/Coartem – 3 days | 1 | Advises continued feeding and fluids for child | 0.5 |
| Asks if the child has a cough | 0.5 | Advises dose of ORS – as often as needed | 0 | Advises to return or take child to facility if worsens | 0.5 |
| Asks if child had convulsions | 0.5 | Gives first dose of amoxicillin | 0 | Checks child health or vaccination record | 0.5 |
| Asks if child had any other problem | 0.5 | Gives first dose of ORS | 0 |  |  |
| Asks if the child has diarrhoea | 0.5 | Gives first dose of zinc | 0 |  |  |
| Asks if child has been vomiting | 0.5 | Prescribes anti-malarial; Coartem | 1 |  |  |
| Asks/checks whether child can drink/feed | 0.5 | Prescribes ORS | 0 |  |  |
| Asks/checks if child is very sleepy/unconsciousness | 0.5 | Advises dose of amoxicillin – 5 days | 0 |  |  |
| Counts breaths of child | 0 | Advises dose of zinc – 10 days | 0 |  |  |
| Performs an RDT (blood test for malaria) on child | 1 | Gives first dose of AL/Coartem | 1 |  |  |
| Checks for chest indrawing in the child | 0 | Gives dose of rectal artesunate/plasmotrim | 0 |  |  |
| Identifies the child as having fast breathing/pneumonia | 0 | Child does not need any treatment from CHW | 0 |  |  |
| Identifies the child as having danger sign/referral symptom or severe illness (any) | 0 | Refers to health facility | 0.5 |  |  |
| Identifies the child as having malaria | 1 | Writes referral note | 0.5 |  |  |
| Identifies the child as having diarrhoea | 0 | Advises use of ITN for child | 0.5 |  |  |
| Prescribes antibiotics; amoxicillin | 0 | Arranges a follow-up visit | 0.5 |  |  |
| Prescribes zinc tablets | 0 | Records visit in CHW registration book | 0.5 |  |  |

**Supplement Table 3. Item weighting – Case study: Beatrice/Janete (severe pneumonia)**

| **Item** | **Score weight** | **Item** | **Score weight** | **Item** | **Score weight** |
| --- | --- | --- | --- | --- | --- |
| Identifies the child as having fast breathing/pneumonia | 1 | Prescribes anti-malarial; Coartem | 0 | Advises continued feeding and fluids for child | 0.5 |
| Identifies the child as having danger sign/referral symptom or severe illness (any) | 1 | Prescribes ORS | 0 | Advises to return or take child to facility if worsens | 0 |
| Identifies the child as having malaria/diarrhoea | 0 | Advises dose of amoxicillin – 5 days | 0 | Checks child health or vaccination record | 0.5 |
| Identifies the child has no major problem | 0 | Advises dose of zinc – 10 days | 0 |  |  |
| Prescribes antibiotics; amoxicillin | 1 | Gives first dose of AL/Coartem | 0 |  |  |
| Prescribes zinc tablets | 0 | Gives dose of rectal artesunate/plasmotrim | 0 |  |  |
| Advises dose of AL/ Coartem – 3 days | 0 | Child does not need any treatment from CHW | 0 |  |  |
| Advises dose of ORS – as often as needed | 0 | Refers to health facility | 1 |  |  |
| Gives first dose of amoxicillin | 1 | Writes referral note | 0.5 |  |  |
| Gives first dose of ORS | 0 | Advises use of ITN for child | 0.5 |  |  |
| Gives first dose of zinc | 0 | Arranges a follow-up visit | 0.5 |  |  |

**Supplement Table 4. Item weighting – Case study: Muteesa/Kizito (severe disease/malaria)**

| **Item** | **Score weight** | **Item** | **Score weight** | **Item** | **Score weight** |
| --- | --- | --- | --- | --- | --- |
| Identifies the child as having fast breathing/pneumonia | 0 | Prescribes anti-malarial; Coartem | 0 | Advises continued feeding and fluids for child | 0.5 |
| Identifies the child as having danger sign/referral symptom or severe illness (any) | 1 | Prescribes ORS | 0 | Advises to return or take child to facility if worsens | 0 |
| Identifies the child as having malaria | 0.5 | Advises dose of amoxicillin – 5 days | 0 | Checks child health or vaccination record | 0.5 |
| Identifies the child as having diarrhoea | 1 | Advises dose of zinc – 10 days | 0 | Advises use of ITN for child | 0.5 |
| Prescribes antibiotics; amoxicillin | 0 | Gives first dose of AL/Coartem | 0 | Arranges a follow-up visit | 0.5 |
| Prescribes zinc tablets | 0 | Gives dose of rectal artesunate/plasmotrim | 1 |  |  |
| Advises dose of AL/ Coartem – 3 days | 0 | Child does not need any treatment from CHW | 0 |  |  |
| Advises dose of ORS – as often as needed | 0 | Refers to health facility | 1 |  |  |
| Gives first dose of amoxicillin | 0 | Writes referral note | 0.5 |  |  |

**Supplement Table 5. Item weighting – Case study: James (severe disease/malaria)**

| **Item** | **Score weight** | **Item** | **Score weight** | **Item** | **Score weight** |
| --- | --- | --- | --- | --- | --- |
| Identifies the child as **NOT** having fast breathing/pneumonia | 1 | Prescribes anti-malarial; Coartem | 0 | Advises continued feeding and fluids for child | 0.5 |
| Identifies the child as **NOT** having danger sign/referral symptom or severe illness (any) | 1 | Prescribes ORS | 0 | Advises to return or take child to facility if worsens | 0.5 |
| Identifies the child as **NOT** having malaria/diarrhoea | 1 | Advises dose of amoxicillin – 5 days | 0 | Checks child health or vaccination record | 0.5 |
| Identifies the child has no major problem | 1 | Advises dose of zinc – 10 days | 0 | Advises use of ITN for child | 0.5 |
| Prescribes antibiotics; amoxicillin | 0 | Gives first dose of AL/Coartem | 0 | Arranges a follow-up visit | 0.5 |
| Prescribes zinc tablets | 0 | Gives dose of rectal artesunate/plasmotrim | 0 |  |  |
| Advises dose of AL/ Coartem – 3 days | 0 | Child does not need any treatment from CHW | 0 |  |  |
| Advises dose of ORS – as often as needed | 0 | Refers to health facility | 0 |  |  |
| Gives first dose of amoxicillin | 0 | Writes referral note | 0 |  |  |
