# Supplementary material for: Improving community health worker treatment for malaria, diarrhoea, and pneumonia in Uganda through inSCALE community and mHealth innovations: A cluster randomised controlled trial
Source: PLOS Digit Health. 2023 Jun 12;2(6):e0000217. doi: 10.1371/journal.pdig.0000217 (PMC10260253; doi:10.1371/journal.pdig.0000217)
Supplement: S2 File — (DOCX) [file pdig.0000217.s002.docx]

# S2 File. Illness and appropriate treatment definitions for fever, diarrhoea and pneumonia

| Illness condition | Illness definition | Appropriate treatment (first line treatments in bold) |
| --- | --- | --- |
| Suspected malaria | Reported fever in the previous four weeks, excluding those confirmed through a blood test as not having malaria. | **Any ACT**: Coartem, Duo-Cotecxin, Amodiaquin-Artesunate, or Artesunate-Fansidar. Rectal Artesunate if malaria + danger sign. Any ACT or Quinine will be analysed as a secondary outcome. |
| Confirmed malaria | Reported fever in the previous four weeks, restricted to those with a positive blood test for malaria |  |
| Diarrhoea | Reported diarrhoea in the previous 4 weeks, specifically three or more watery stools in a 24hr period. | **ORS** (homemade or ready-made). ORS plus zinc supplementation will be analysed as a secondary outcome. |
| Suspected pneumonia (“pneumonia” in this report) | Reported cough with fast/difficult breathing (which was not due to a blocked nose), or chest indrawing | Amoxicillin, Chloramphenicol, Erythromycin, Ceftriaxone, Azythromycin or Cephalexin |
| Episode of any of suspected malaria, diarrhoea or suspected pneumonia | Each episode where one of the above sets of signs occurs is viewed as a separate case |  |

Note: As co-morbidities occur in this setting, no diagnostic hierarchy was assigned.
