# Supplementary material for: Improving community health worker treatment for malaria, diarrhoea, and pneumonia in Uganda through inSCALE community and mHealth innovations: A cluster randomised controlled trial
Source: PLOS Digit Health. 2023 Jun 12;2(6):e0000217. doi: 10.1371/journal.pdig.0000217 (PMC10260253; doi:10.1371/journal.pdig.0000217)
Supplement: S3 File — (DOCX) [file pdig.0000217.s003.docx]

**S3 File. Appropriate treatment results based on whole child analysis**

This is defined as the proportion of children with MDP who received appropriate treatment for all conditions (out of these three) they concurrently presented with in the exposure period.

| **Indicator** | **CONTROL** | **VHC** | | **mHEALTH** | |
| --- | --- | --- | --- | --- | --- |
|  | % (N) | % (N) | Versus Control  RR (95% CI) P | % (N) | Versus Control  RR (95% CI) P |
| Appropriate treatment | 54.55 (516) | 61.70 (604) | 1.12 (1.02-1.24) 0.021 | 63.34 (558) | 1.13 (1.02-1.26)  0.017 |
| Appropriate treatment (ORS + Zinc for diarrhoea) | 52.64 (498) | 56.08 (549) | 1.07 (0.97-1.17) 0.166 | 60.16 (530) | 1.13 (1.02-1.24)  0.014 |
| Appropriate treatment (first line drugs only) | 52.75 (499) | 59.96 (587) | 1.13 (1.02-1.25) 0.022 | 61.18 (539) | 1.13 (1.01-1.25)  0.031 |
