# Supplementary material for: Improving community health worker treatment for malaria, diarrhoea, and pneumonia in Uganda through inSCALE community and mHealth innovations: A cluster randomised controlled trial
Source: PLOS Digit Health. 2023 Jun 12;2(6):e0000217. doi: 10.1371/journal.pdig.0000217 (PMC10260253; doi:10.1371/journal.pdig.0000217)
Supplement: S4 File — (DOCX) [file pdig.0000217.s004.docx]

# S4 File. Baseline-endline change in appropriate treatment and CHW care seeking across inSCALE arms (difference-in-difference analysis)

|  | **Control arm** | **VHC Arm** | | | **mHealth Arm** | | |
| --- | --- | --- | --- | --- | --- | --- | --- |
|  | **Risk difference (SD)** | **Risk difference (SD)** | **Risk difference in difference** | **p** | **Risk difference (SD)** | **Risk difference in difference** | **p** |
| Cluster mean Δ in appropriately treated episodes: endline - baseline (percentage units) | +13.9% (12.13) | +20.1% (11.16) | +6.1  (-3.0–15.2) | 0.182 | +18.8%  (11.08) | +4.9  (-4.3–14.0) | 0.288 |
| Cluster mean Δ in CHW care-seeking: endline - baseline (percentage units) | +14.9% (15.5) | +10.1% (16.0) | -4.7  (-17.7–8.2) | 0.467 | +21.2  (17.5) | +6.3  (-6.7–19.3) | 0.331 |
