# Supplementary material for: Improving community health worker treatment for malaria, diarrhoea, and pneumonia in Uganda through inSCALE community and mHealth innovations: A cluster randomised controlled trial
Source: PLOS Digit Health. 2023 Jun 12;2(6):e0000217. doi: 10.1371/journal.pdig.0000217 (PMC10260253; doi:10.1371/journal.pdig.0000217)
Supplement: Attachment — Submitted filename: feedback review.docx [file pdig.0000217.s005.docx]

This is cluster randomized trial conducted in Uganda where the authors reported that The inSCALE mHealth and VHC interventions have the potential to reduce CHW attrition and improve the care quality for sick children. However, there are some caveats that need to be addressed.

1. The sample size was known only after the study was done. Can the authors provide the sample size calculation with different ingredients used for its estimate?
2. In the methods section
   a. There was a suggestion of using only random effect model (Statistical analysis, line 280) for the multivariate model. However, in table 5, both random and fixed effect were used.
   b. Also, can the authors provide rational of using intention to treat analysis (line 277, statistical analysis) and not per protocol or both?
3. In the limitation section (page 28), one will wonder if contamination cases between study arms were not found.
4. On page 28, last two sentences, it is mentioned that the improvements observed in the intervention arms occurred despite problems with drug stocks and other programmatic challenges. Can the author specify the type of programmatic challenges?
5. Also in the conclusion section, one would expect to see the study perspectives or future. What can the authors suggest from this study in that regard?
6. Line 267, there was an typos error.
