# Supplementary material for: Improving community health worker treatment for malaria, diarrhoea, and pneumonia in Uganda through inSCALE community and mHealth innovations: A cluster randomised controlled trial
Source: PLOS Digit Health. 2023 Jun 12;2(6):e0000217. doi: 10.1371/journal.pdig.0000217 (PMC10260253; doi:10.1371/journal.pdig.0000217)
Supplement: Attachment — Submitted filename: Reviewers comments and responses. docx.docx [file pdig.0000217.s006.docx]

Reviewer #1: This is cluster randomized trial conducted in Uganda where the authors reported that The inSCALE mHealth and VHC interventions have the potential to reduce CHW attrition and improve the care quality for sick children. However, there are some caveats that need to be addressed.

1.	*The sample size was known only after the study was done. Can the authors provide the sample size calculation with different ingredients used for its estimate?*

**Authors’ response**: Sample size information has now been added to the new sub-section “Sample Size” in the methods section on Page 15. The added section reads as follows:

Sample sizes were based on the primary outcome, namely appropriate treatment of cases with suspected malaria, diarrhoea, and pneumonia. Procedures for calculating sample size are described in full in the study protocol paper [14]. In brief, assuming a control group percentage of appropriate treatment of 47% episodes of MDP and a coefficient of variation of 0.17 (values estimated from baseline data) gave us 90% power to detect a 15% change in appropriate treatment as a result of either intervention, sampling 155 children in each of 39 clusters. This sample size would also enable us to detect changes of 15%-25% in appropriate treatment of reported malaria, diarrhoea, and pneumonia individually with power of at least 80% [14].

2.	*In the methods section*

*a.	There was a suggestion of using only random effect model (Statistical analysis, line 280) for the multivariate model. However, in table 5, both random and fixed effect were used.*

**Authors’ response**: The analysis approach with respect to fixed and random effects has been better clarified in the Analysis sub-section on Page 15. The section now reads as follows:

All analyses were intention-to-treat and accounted for the cluster-randomised design. This was achieved by inclusion of the variables on which the randomisation was restricted at baseline as fixed effects (appropriate treatment for MDP, health worker motivation and household log cost of care seeking), and specifying random-intercepts for sub-county cluster units for either binary or continuous outcomes.

b.	*Also, can the authors provide rational of using intention to treat analysis (line 277, statistical analysis) and not per protocol or both?*

**Authors’ response**: Thank you for this important question. We carried out an intention to treat analysis because this is the recommended primary approach for reporting of randomised trial impact outcomes, as outlined in the CONSORT statement and checklist which we were required to submit along with this manuscript. See here: <http://www.consort-statement.org/checklists/view/32--consort-2010/96-statistical-methods>).

We have also shown the impact of the intervention in a subset of children who were taken to the community health worker or to a public health facility (Table 7) – though not all children accessed these providers, these were the target providers within which the intervention was implemented; this provider-based analysis is one form of per protocol analysis.

We are however keen to explore the relationship between intervention implementation strength and impact outcomes – such analyses are outside the scope of this impact paper but will be revisited in future papers. We have added wording to the Discussion section to this effect on page 28.

3.	*In the limitation section (page 28), one will wonder if contamination cases between study arms were not found.*

**Authors’ response:** Thank you for this comment. A sentence has been added in the discussion on page 30, mentioning the reason why we believe contamination was minimal. The section now reads as follows:

Our study limitations included the delivery of the interventions in only one area of Uganda limiting study generalisability across contexts. While contamination between study arms is possible, it was likely minimal, given that community members tend to use the CHW who they have elected for their community.

4.	*On page 28, last two sentences, it is mentioned that the improvements observed in the intervention arms occurred despite problems with drug stocks and other programmatic challenges. Can the author specify the type of programmatic challenges?*

**Authors’ response**: A small edit has been done to the sentence to address this comment (now on page 30). The sentence now reads as follows:

The improvements observed in the intervention arms occurred despite programmatic challenges such as drug stockouts and irregular supervision; barriers which these interventions could not overcome.

*5.	Also in the conclusion section, one would expect to see the study perspectives or future. What can the authors suggest from this study in that regard?*

**Authors’ response:** Thanks for this comment. We have added a blurb on future directions in the conclusions on page 30-31. The section now reads as follows:

We recommend that future studies aiming to evaluate drivers of CHW performance and motivation further try to unpack the mechanisms of action, as well as the care seeking pathways among caregivers who did and did not seek care from a CHW, to understand their motivations. We also see a lot of value in evaluating a combination of aspects from both the VHC and mHealth intervention, as these approaches might have complementary benefits. A combined VHC-mHealth intervention would also more closely reflect the reality of today's CHW programmes, which increasingly adopt digital health tools for CHW task management, data reporting and eLearning, in combination with community mobilisation activities to optimise CHW management.

*6.	Line 267, there was an typos error.*

**Authors’ response:** Noted, thanks!

*Reviewer #2: This manuscript presented the results of a large cluster randomized trial. Importantly, process evaluation results are presented alongside the main trial findings. While this increases the manuscripts complexity, this combination helps the reader to more fully understand the trial results in context. My primary comment lies in the description of the interventions and trial arms. Other comments that strengthen results reporting are also listed below.*

*Abstract – the number of groups is not quite clear in the abstract. Says two interventions, but later on there are three arms (mHealth; VHC or usual care (control) arms)? It is understood once the reader reads the paper, but I think it should be more clear up front*.

**Authors’ response**: Thanks for pointing this out. We have clarified this in the abstract, which now reads as follows:

The inSCALE cluster randomised controlled trial evaluated two interventions, mHealth and Village Health Clubs (VHCs) which aimed to improve Community Health Worker (CHW) treatment for malaria, diarrhoea, and pneumonia within the Integrated Community Case Management (iCCM) programme. The interventions were compared with standard care in a control arm.

*Mechanism of improved CHW management presented in Interpretation is not well referenced in the Results section*

**Authors’ response**: We have made some edits to the interpretation to clarify our message.

Introduction

*Paragraph starting on line 127 describes the two approaches to increasing CHW engagement and effectiveness and they are presented as individual approaches. However, it is unclear to this reader why these two approaches are not complementary or could not be combined into a package approach. Meaning, what was the rationale for testing these approaches separately rather than together? Furthermore, Line 146 seems to indicate they have the same potential effects.*

**Authors’ response**: While programmatically, it would make sense to combine aspects from both the VHC and mHealth intervention, this study set out to evaluate these two approaches separately against standard care in order to isolate their respective independent impact. The combined impact of the two approaches would have required a much larger study with a more complex study design. As such, we did not attempt to compare the two interventions against each other, but rather aim to establish whether motivation, performance and retention can be achieved using different approaches that address the similar pathways to CHW motivation (see another publication describing the pathway to develop the interventions based on formative research (<https://human-resources-health.biomedcentral.com/articles/10.1186/s12960-015-0020-8>). We have added a sentence to address this at the end of the discussion on page 30, which now reads as follows:

We recommend that future studies aiming to evaluate drivers of CHW performance and motivation further try to unpack the mechanisms of action, as well as the care seeking pathways among caregivers who did and did not seek care from a CHW, to understand their motivations. We also see a lot of value in evaluating a combination of aspects from both the VHC and mHealth intervention, as these approaches might have complementary benefits. A combined VHC-mHealth intervention would also more closely reflect the reality of today's CHW programmes, which increasingly adopt digital health tools for CHW task management, data reporting and eLearning, in combination with community mobilisation activities to optimise CHW management.

*Line 143 The Innovations at Scale for Community Access and Lasting Effects (inSCALE) project set out to develop and evaluate two interventions based on the social identity*

*Please clarify the phrase “…based on the social identity…”*

**Authors’ response:** Thanks for noting that error. We have added “social identity approach described above”.

Methods

*Table 1. Unclear what is meant by the phrase “and patrons” in the phrase “Supervisor support and patrons”.*

*Some overlap, such as supervisor support among the entries. Suggest review of each row and merging them (not separating by mHealth solutions, village health clubs, CHW program support)*

**Authors’ response:** Thanks for this comment. We have revised table 1 to make it clearer and with less overlap.

*Page 17, line 291: This is not clear – is this referring to the scales introduced in line 195? Why were these methods applied to the scales used?*

**Authors’ response:**

Yes, these methods were applied to the CHW motivation and social identification measurement scales to increase confidence in their validity for use with the target population. The methods are consistent with the robust approaches advocated in the literature for development of scales for use with new populations. These include qualitative measures to check terms and wording mean what they are intended to mean for respondents, and quantitative methods to reduce extraneous items and develop the most reliable and parsimonious scales. The wording in the paragraph has been edited to make this clearer. We acknowledge that the initial wording implied that the scale validation methods were used to generate motivation and social identification scores when, in fact, they were used to create valid and reliable scales for generating these scores. Edits were made on page 9 and 16 to add further clarity on this.

Results

*Fig 1, please add abbreviations under table. Suggest match Arm names the names in the manuscript for consistency*

**Authors’ response:** Thanks for noting this error. This has been corrected.

*Page 22 – consider adding “whole child” concept to Methods section*

**Authors’ response:** Thanks for this suggestion. We have added a sentence on this on page 14 in the methods section. The sentence now reads as follows:

….and viii) appropriate treatment of a sick child or “whole child analysis”, where each child was categorised as appropriately treated if they received the correct treatment for all reported episodes of MDP occurring the last 4 weeks and not appropriately treated if they were not correctly treated for one or more episode occurring in that period.

*Provide scale ranges for all variables as footnotes (e.g., Table 6)*

**Authors’ response:** We have now provided the scale ranges for fixed-score-based outcomes in Table 6 (Page 23 Results)

Discussion

*I would appreciate a larger discussion of process evaluation that you would consider adding to future work to elucidate some of the unknowns behind the mechanism of action. For example, interviews with caregivers who did and did not seek care to understand their motivations*

**Authors’ response:** Point taken, and as per our response to Reviewer 1’s comment number 5, we have added a blurb at the end of the discussion on pages 30-31 that comments on future work. The conclusion now reads as follows:

We recommend that future studies aiming to evaluate drivers of CHW performance and motivation further try to unpack the mechanisms of action, as well as the care seeking pathways among caregivers who did and did not seek care from a CHW, to understand their motivations. We also see a lot of value in evaluating a combination of aspects from both the VHC and mHealth intervention, as these approaches might have complementary benefits. A combined VHC-mHealth intervention would also more closely reflect the reality of today's CHW programmes, which increasingly adopt digital health tools for CHW task management, data reporting and eLearning, in combination with community mobilisation activities to optimise CHW management.
