# Supplementary material for: Improving community health worker treatment for malaria, diarrhoea, and pneumonia in Uganda through inSCALE community and mHealth innovations: A cluster randomised controlled trial
Source: PLOS Digit Health. 2023 Jun 12;2(6):e0000217. doi: 10.1371/journal.pdig.0000217 (PMC10260253; doi:10.1371/journal.pdig.0000217)
Supplement: Attachment — Submitted filename: Rebuttal letter.docx [file pdig.0000217.s007.docx]

**Rebuttal letter**

We have responded to final reviewer requests as follows:

1. Reviewer #2: The authors have done a sufficient job responding to both sets of reviewers' comments. However, there is one in which I still have a comment: 
 
Provide scale ranges for all variables as footnotes (e.g., Table 6) 
Authors’ response: We have now provided the scale ranges for fixed-score-based outcomes in Table 6 (Page 23 Results).  
 
The request was to provide possible scale ranges for all variables as footnotes, e.g., motivation score 1 (low) to 10 (high). I believe the authors have added the maximum observed value in the table 5 itself.

***Author response***: Apologies for the misunderstanding. We have now provided the possible scale ranges in table 6. Given that motivation and social identity was calculated using factor analysis, we had to convert the scores back to raw scores to be able to present the scale ranges. We have therefore updated the table with the raw scores for both motivation and social identity. The conclusions remain unchanged so this edit has negligible implications on the findings.

2. Journal requirement edits: Please review your reference list to ensure that it is complete and correct.

***Author response***: We have deleted reference nr 17 which was referring to a paper that is still not published.
